# The analysis of pedestrian flow in the smart city by improved DWA with robot assistance

**DOI:** 10.1038/s41598-024-62134-8

**Published:** 2024-05-20

**Authors:** Yingyue Hu, Huizhen Long, Min Chen

**Affiliations:** 1 School of Government, Central University of Finance and Economics, Beijing, 10081 China; 2 Strategic Planning Office, Wuhan Business University, Wuhan, China; 3 School of Tourism and Hospitality Management, Hong Kong Polytechnic University, Hung Hom, Hong Kong; 4 School of Cultural Tourism and Geography, Guangdong University of Finance and Economics, Guangzhou, China; 5 School of Business, Wenzhou University, Wenzhou, China

**Keywords:** Dynamic window approach, Simulation experiment, Pedestrian traffic flow, Regulation of pedestrian flow, Smart city, Computational science, Computer science, Information technology, Scientific data

## Abstract

With the acceleration of urbanization in China, the urban population continues to grow, leading to frequent occurrences of crowded public spaces, which in turn trigger traffic congestion and even safety accidents. In order to more effectively control pedestrian flow, enhance the efficiency and safety of public spaces, this experiment conducts in-depth research and improvement on the traditional Dynamic Window Approach (DWA), and applies it to the fine control of pedestrian flow. Specifically, this study comprehensively reviews and analyzes the characteristics of pedestrian traffic flow and the working principles of traditional DWA. Based on this, the shortcomings of traditional DWA in dealing with complex pedestrian flow scenarios are identified, and targeted improvement solutions are proposed. The core of this improvement scheme lies in the introduction of a new evaluation function, enabling DWA to more accurately balance various factors in the decision-making process, including pedestrian movement speed, direction, and spatial distribution. Subsequently, the improved DWA is validated through simulation experiments. The experimental scenario is set in an area of 18 m*18 m, and compared with traditional DWA, the improved DWA shows significant advantages in trajectory length and travel time. Specifically, the trajectory length of the traditional DWA robot is 19.4 m, with a required time of 34.8 s, while the trajectory length of the improved DWA robot is shortened to 18.7 m, and the time is reduced to 18.6 s. This result fully demonstrates the effectiveness of the improved DWA in optimizing pedestrian flow control. The improved DWA proposed in this study not only has strong scientific validity but also demonstrates high efficiency in practical applications. This study has important reference value for improving the safety of urban public spaces and improving pedestrian traffic flow conditions, and provides new ideas for the further development of pedestrian flow control technology in the future.

## Introduction

As the oldest and most basic way of travel for human beings, walking is not only the initial and final part of various modern modes of transportation, but also the main way for people to act when shopping, visiting, tourism, and other activities, and plays an important role in daily life. According to statistics, walking accounts for 40–60% of all travel methods in urban domestic and international transportation, and people's walking time accounts for more than 20% of the overall travel time, which is of great practical significance and broad scope of application for the study of pedestrian flow^1^.

With the increase of the urban population and the number of travels caused by urbanization in China, coupled with the construction of transportation hubs and activity centers, dense crowds are bound to form in public activity places, causing traffic congestion. If there is an emergency, it will cause the crowd to panic so that there will be a stampede, causing huge loss of life and property. On July 24, 2010, at the E-Music Festival in Irisburg, Germany, a panic stampede caused by congestion in underground passages, killing 21 people and injuring 342 others. On December 31, 2014, a pedestrian fell on the steps on the Bund in Shanghai and caused a serious stampede, resulting in 36 deaths and 49 injuries^2,3^. Therefore, in these scenarios, finding the basic rules of pedestrian flow and effectively controlling the circulation and evacuation of pedestrians are crucial to optimizing traffic efficiency, avoiding accidents, and building a safe, comfortable, and fast pedestrian traffic system. The study of intelligent equipment to optimize the transportation system plays an important role in the development of cities, and can also contribute to the progress of smart cities in the future.

To ensure the safety of pedestrian flow, it is necessary to reasonably regulate pedestrian flow. The traditional Dynamic Window Approach (DWA) is improved, and it is applied to the regulation of pedestrian flow. Based on this, firstly, the pedestrian traffic flow and the traditional DWA are studied. Secondly, the problems are improved and added, and an improved DWA is proposed. Finally, it is proved that the proposed improved DWA algorithm is scientific and effective for the regulation of pedestrian flow through simulation experiments. The contributions of this research include: 1) It has certain reference significance for the regulation of pedestrian flow in smart cities; 2) It provides vital technical support for the improvement of urban transportation; 3) It contributes to the construction and progress of smart cities.

## The regulation of pedestrian flow by improved DWA

### Research summary

With the change of society, the construction of smart cities has become the main goal of urban construction. However, the current urban transportation work is not perfect enough to meet the requirements of smart cities. Therefore, more research is needed to provide the impetus for the development of intelligent transportation. Although the development of command transportation is not perfect, many studies have provided technical support for it. Zhao et al. (2022) pointed out that with the rapid expansion of the current society, people's work and life are increasingly inseparable from transportation. The establishment of fast transportation requires the development of an intelligent transportation system as soon as possible to alleviate the congestion and blockage of the entire urban traffic flow^4^. Putra et al.^5^ proposed that the development of information and communication technology has promoted the construction of smart transportation, which will further improve the accessibility of urban transportation and affect the travel of residents. Under the background of the global consensus on addressing climate change and reducing carbon emissions, due to the complex influence mechanism among "smart transportation-accessibility-travel behavior (carbon emission)", it is difficult to judge whether the improvement of urban transportation accessibility under the background of intelligence will increase or reduce the carbon emission of travel^5^. Zhao et al.^6^ proposed that in order for mobile robots to work efficiently in various environments, it is necessary to select an appropriate path planning algorithm according to the actual terrain. To this end, a hybrid path planning algorithm based on the optimized DWA algorithm was introduced, and four typical terrains of U-shaped, S-shaped, L-shaped, and narrow channels were built in the simulation environment for pathfinding experiments. Meanwhile, the weight recursive formula for mapping is improved to eliminate the dependence on the data of the previous moment, and the efficiency of the algorithm is improved^6^. Ren et al.^7^ put forward an improved DWA obstacle avoidance algorithm based on fuzzy reasoning, that is, based on the conventional speed window, a perception window based on onboard sensors is designed, and a dual-window model is formed to further optimize the constrained speed space. According to the distribution of obstacles, the weights of the evaluation function are dynamically adjusted. The research results have obvious scientific value and practical significance for the development of autonomous obstacle avoidance technology for unmanned boats^7^.

In summary, the current development of smart cities is crucial. However, the existing traffic conditions are not satisfactory, hence more research is urgently needed to provide technical support and references. The emergence of the DWA obstacle avoidance algorithm provides an important technical foundation for this goal. By optimizing DWA, strong driving force can be provided for its application in traffic construction. In the optimization process, particular attention was paid to the improvement of the formula, by introducing new evaluation functions and optimizing decision-making mechanisms, making the DWA algorithm more efficient and accurate in dealing with complex traffic environments. These improvements not only enhance the performance of DWA but also further promote the development of smart city traffic construction.

### Pedestrian traffic flow

Since the 1930s, the theory of pedestrian flow has gradually entered the research scope of scholars in domestic and overseas. British scholars first put forward the pedestrian flow theory in 1958 and studied pedestrian flow in London's underground passage^8^. With the improvement of the mathematical system and the development of computer technology, the study of pedestrian flow has developed rapidly in information collection, theoretical modeling and simulation analysis. Early information collection mainly took videos and then manually counted points. Thanks to the development of image recognition theory and technology, pedestrian speed, flow and other information can be measured in real time, and the accuracy has greatly increased^9^. In terms of pedestrian flow modeling, it can be divided into microscopic, macroscopic and mesoscopic models from different perspectives according to its characteristics.

The microscopic model of pedestrian flow treats each pedestrian as an independent individual, and mainly considers the relationship between the individual and other pedestrians or obstacles. Generally, it is divided into two categories according to the modelling mechanism. The first category is mechanics-based models, such as Social Force Model Social Force Model (SFM). The second category is rule-based models, such as Cellular Automata (CA) model.

**Figure 1:**
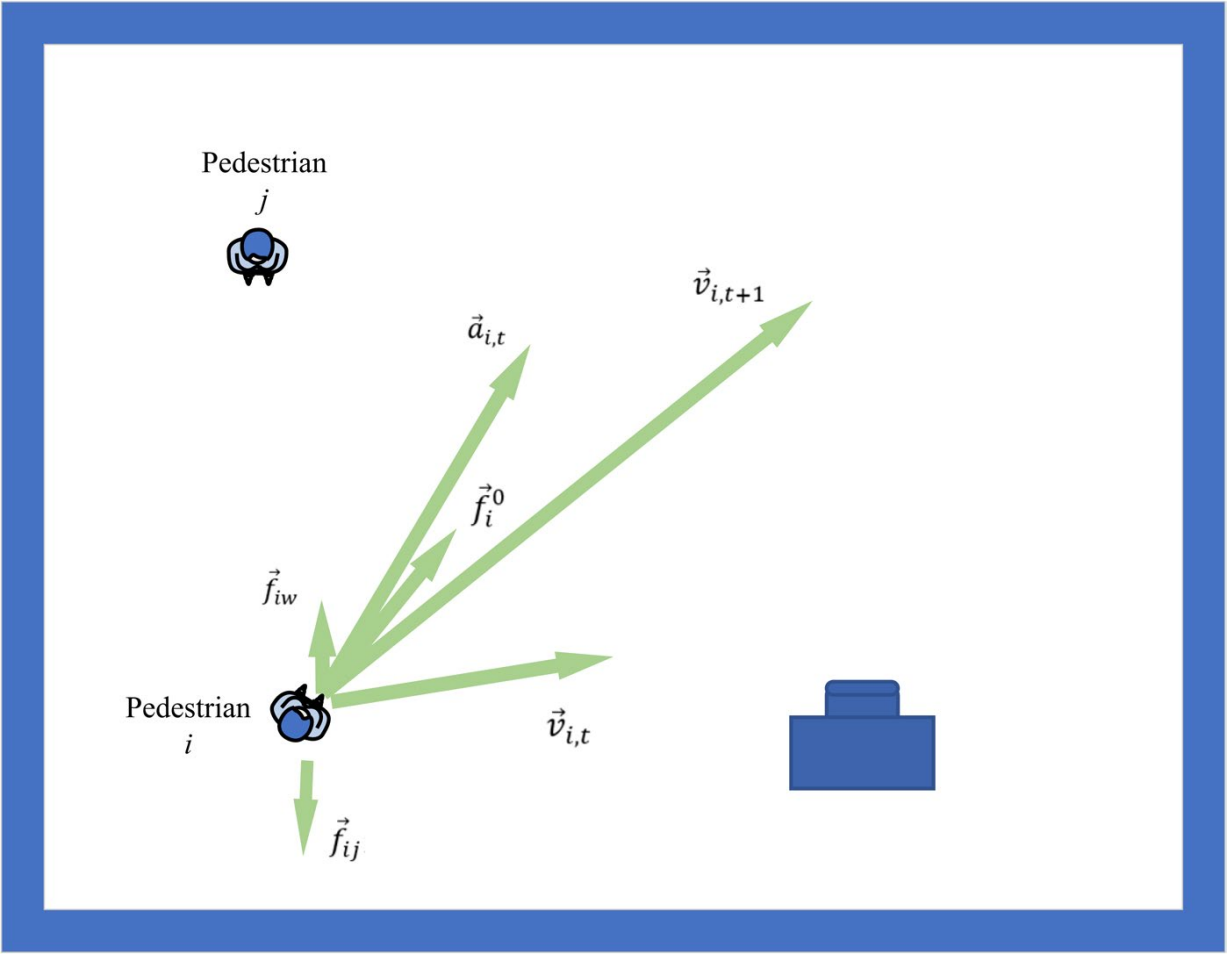
The force diagram of the SFM.

The SFM was born in 1995^10^. In this model, pedestrians are mainly affected by their own driving force, the interaction force of moving pedestrians, and the force of static obstacles such as walls, as shown in Fig. 1.

In Fig. 1, ${\overrightarrow{f}}_{i}^{0}$ is the driving force of pedestrian $i$ towards the target point. ${\overrightarrow{f}}_{ij}$ is the force of pedestrian $i$ from pedestrian $j$, and ${\overrightarrow{f}}_{iw}$ is the force of pedestrian $i$ from obstacle $w$. Thus, the acceleration ${\overrightarrow{a}}_{i,t}$ of pedestrian $i$ at time $t$ is shown in Eq. (1):

1 $${\overrightarrow{a}}_{i,t}=\frac{{\overrightarrow{f}}_{i}^{0}+\sum_{j(j\ne i)} {\overrightarrow{f}}_{ij}+\sum_{w} {\overrightarrow{f}}_{iw}}{{m}_{i}}$$

In Eq. (1), ${m}_{i}$ is the mass of pedestrian $i$. According to the speed ${\overrightarrow{v}}_{i,t}$ of pedestrian $i$ at time $t$, the speed ${\overrightarrow{v}}_{i,t+1}$ at the next moment is obtained. The calculation is shown in Eq. (2):

2 $${\overrightarrow{v}}_{i,t+1}={\overrightarrow{v}}_{i,t}+{\overrightarrow{a}}_{i,t}$$

In recent years, foreign scholars have analyzed the influence of passageway structure on evacuation efficiency by simulating the escape process of pedestrians with different panic levels. Considering the impact of pedestrian moving speed on the repulsive force, a generalized centrifugal model is proposed^11,12^. Domestic scholars have simulated the escape phenomenon when most pedestrians are not familiar with the structure of the evacuation passageway through the SFM^13^.

The SFM can better simulate the self-organizing phenomena of pedestrian flow, such as blockage, formation, and oscillating flow, and can fully consider the psychological state of pedestrians, information perception, conformity or dependence and other factors. Therefore, it has been widely developed and applied, but it also has a large amount of computation. When the description of the collision phenomenon is not well, it is easy to fall into local optimum and other disadvantages.

The CA model was proposed in 1987 as a dynamic model that is discrete in time, space and state^14^. The model evolved from the Lattice-Gas Automata, dispersing pedestrians and obstacles into grid structures, with a cell inside each grid. Each cell is either empty, occupied by pedestrians, or occupied by obstacles. Each pedestrian can move around in 8 directions at a time. Each CA consists of a background field superimposed by a static field and a dynamic field, usually described by a 3*3 matrix. The value in each grid in the background field is the probability of pedestrians moving to the cell. At each time step, the motion of all pedestrians is updated in parallel mode^15,16^. In the background field, the probability of pedestrian moving to the cell $(i,j)$ is shown in Eq. (3):

3 $${M}_{ij}=N{\text{exp}}\left({k}_{D}{D}_{ij}\right){\text{exp}}\left({k}_{S}{S}_{ij}\right)\left(1-{n}_{ij}\right){\xi }_{ij}$$

$N$ is the normalization coefficient, which ensures that the sum of the probabilities of moving in all directions is 1. Its value is shown in Eq. (4):

4 $$N={\left[\sum_{i,j} {\text{exp}}\left({k}_{D}{D}_{ij}\right){\text{exp}}\left({k}_{S}{S}_{ij}\right)\left(1-{n}_{i,j}\right){\xi }_{ij}\right]}^{-1}$$

${S}_{ij}$ and ${D}_{ij}$ represent the values of the static and dynamic fields at the cell $(i,j)$, respectively.${k}_{S}$ and ${k}_{D}$ show the coupling strengths of the static and dynamic fields, respectively. When the cell $(i,j)$ is not occupied by other pedestrians, the value of ${n}_{ij}$ is 0, otherwise, it is 1. When the cell $(i,j)$ is not occupied by an obstacle, the value of ${\xi }_{ij}$ is 0, otherwise, it is 1.

By combining the CA model with the SFM, the simulation analysis of pedestrian evacuation in a single exit passageway can be performed^17^. In 2015, a multi-speed CA model was proposed. By investigating the proportion of pedestrians with different speeds, the impact on the overall evacuation efficiency was studied^18^. In 2017, the CA model was applied to study the pedestrian motion characteristics at the entrances and exits of a comprehensive transfer passageway^19^.

The CA model can effectively simulate the influence of different pedestrian psychology, density and movement direction on the circulation efficiency under different facility structures through different update rules. It is easy to implement and has a high operation speed, and is a relatively mainstream microscopic model. However, it has the defects of too simple rules, rough description of individual behavior, and limited moving speed by grid.

The macroscopic model of pedestrian flow takes the whole pedestrian as the research object, and does not consider the information such as force, psychology and movement of individual pedestrians, and describes the movement of pedestrian flow as the change of macroscopic characteristics such as pedestrian density, speed and flow over time and space. When the simulation of the microscopic model reaches a certain scale, extracting its macroscopic features can also achieve similar effects as the macroscopic model, and the description method is more refined. At present, the representative macroscopic models include fluid mechanics model, path planning model and network flow model.

The fluid mechanics model was born in 1971^20^. The model compares the pedestrian flow to a fluid, and believes that the characteristics of pedestrian movement are similar to gas molecules at lower density, but similar to liquid molecules at higher density. The relevant theory of fluid mechanics is used to analyze the movement of pedestrian flow. In 2000, some scholars regarded pedestrian flow as a continuous fluid medium, and considered its thinking and selection behavior in the movement of pedestrian flow. They thought that pedestrians would choose the path that can reach the destination while the movement cost is the least. The cost function considers both the minimization of movement time and the avoidance of entering high-density areas, and plays a milestone role in the development of macroscopic models of pedestrian flow^21^. The model considers that the flow, density and speed of pedestrians meet Eq. (5):

5 $$\frac{\partial \rho }{\partial t}+\frac{\partial (\rho u)}{\partial x}+\frac{\partial (\rho v)}{\partial y}=0$$

In Eq. (5), $\rho$ is the density of pedestrians, and $u$ and $v$ are the partial speed of pedestrians in the $x$ and $y$ directions, respectively.

In 2010, a reactive-predictive fluid mechanics model was proposed to study the obstacle avoidance problem of pedestrian flow based on mixed strategies^22^. A high-order fluid mechanics model was constructed in 2015 considering pedestrian acceleration and inertia. After decades of development, the fluid mechanics model can better describe various macroscopic characteristics of pedestrian flow, and can describe some microscopic characteristics such as pedestrian path selection, acceleration, and steering^23^. However, it has the disadvantages of a large number of equations, complex forms, and difficulty in solving.

The research scale of the mesoscopic model of pedestrian flow is between the macro model and the micro model. It does not take all pedestrians as a whole, and does not take too much into account the interaction between individual pedestrians. The object of its analysis is a pedestrian group with similar characteristics such as density and speed. The model is generally composed of a pedestrian group and a potential field or grid that describes the environment in which it is located. The most representative mesoscopic model is aerodynamics, which was established in 1998. It considers pedestrian awareness, expected speed, and the interaction between pedestrians, and describes some common pedestrian phenomena such as congestion, queuing, and crossing^24^. The mesoscopic model can take advantage of a small amount of calculation in the macroscopic model and the consideration of individual differences in the microscopic model, but also has the disadvantage that it cannot accurately describe the characteristics of individual pedestrians and explain macroscopic phenomena.

### Traditional DWA

The DWA is a local path planning algorithm based on trajectory prediction, which determines the velocity at the next moment by computing the score of each predicted trajectory. DWA has advantages such as low computational complexity, good obstacle avoidance effect, and strong algorithm flexibility. In DWA, the analysis of the robot's motion model is required for velocity space sampling and trajectory estimation, while trajectory scoring requires setting appropriate evaluation functions and parameters according to different needs^25,26^. Improvements to DWA focus on optimizing the formulas. Firstly, the evaluation function is improved to more accurately assess the quality of different trajectories, thereby selecting more suitable motion paths. Secondly, the method of velocity space sampling is optimized to improve sampling efficiency and accuracy. Additionally, improvements are made to the trajectory estimation algorithm to better adapt to complex and changing traffic environments. Through these improvements, the performance and stability of DWA are successfully enhanced, enabling it to better handle various traffic scenarios and provide strong technical support for the construction of smart cities.

For each time $t$, the robot has two parameters, linear velocity ${v}_{t}$ and angular velocity ${\omega }_{t}$. When calculating the trajectory of the robot, the time interval between three adjacent moments is $\Delta t$. In $\Delta t$, the linear velocity of the robot is considered to be constant, and the angular velocity is 0. The displacement and angle of the robot in the coordinate system within $\Delta t$ are shown in Eq. (6):

6 $$\left\{\begin{array}{l}\Delta x={v}_{t}\Delta tcos\left({\theta }_{t}\right)\\ \Delta y={v}_{t}\Delta tsin\left({\theta }_{t}\right)\\ \Delta \theta =\Delta t{\omega }_{t}\end{array}\right.$$

(${v}_{t0}$,${\omega }_{t0}$) remains unchanged. After time $T$, the displacement and angle of the robot are shown in Eq. (7):

7 $$\left\{\begin{array}{l}{x}_{{t}_{0}+T}={x}_{{t}_{0}}+\sum_{t={t}_{0}}^{{t}_{0}+T} {v}_{{t}_{0}}\Delta tcos\left({\theta }_{t}\right)\\ {y}_{{t}_{0}+T}={y}_{{t}_{0}}+\sum_{t={t}_{0}}^{{t}_{0}+T} {v}_{{t}_{0}}\Delta tsin\left({\theta }_{t}\right)\\ {\theta }_{{t}_{0}+T}={\theta }_{{t}_{0}}+{\omega }_{{t}_{0}}T\end{array}\right.$$

The velocity space sampling of the robot is to determine the range of the velocity (${v}_{t+1}$,${\omega }_{t+1}$) at the next moment according to the current velocity of the robot (${v}_{t}$,${\omega }_{t}$), and the process of uniform sampling in this range to get the feasible speed of the array. When determining the range, the velocity of the robot cannot exceed its maximum value, and the velocity change of the robot per unit time cannot exceed its maximum value. The sampling steps are set according to the required precision. The angular velocity and the linear velocity and the two dimensions are uniformly sampled in the value space, and then combined with each other, the data (${v}_{t+1}$,${\omega }_{t+1}$) can be obtained.

**Figure 2:**
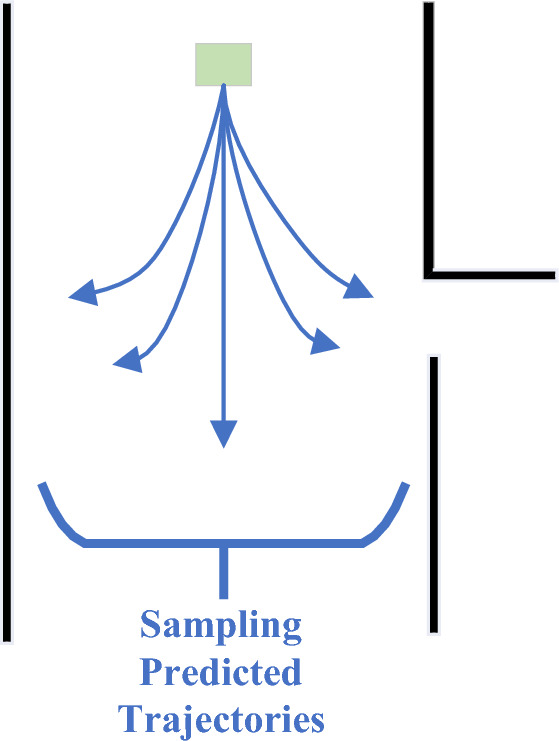
Schematic diagram of sampling predicted trajectories.

After the sampling space is established, the time $T$ is set. According to Eq. (7), the predicted path of each velocity combination can be obtained. The velocity (${v}_{t}$,${\omega }_{t}$) remains unchanged, and the trajectories of the robot in the subsequent time $T$ are shown in Fig. 2.

After the predicted trajectories are obtained, they will be evaluated. In the traditional DWA, the evaluation function is shown in Eq. (8):

8 $$J(v,\omega )=\sigma [\alpha \cdot \text{heading }(v,\omega )+\beta \cdot \text{obdist }(v,\omega )+\gamma \cdot \text{ velocity }(v,\omega )]$$

In Eq. (8), three sub-evaluation functions and some coefficients are included. In $\text{heading }(v,\omega )$, it calculates the difference $\theta$ between the heading angle of the robot at the end of the predicted trajectory and the angle between the robot position and the heading angle of the target point, to evaluate the degree of the trajectory toward the target point. The smaller the angle difference $\theta$, the higher the score. $\alpha$ is the weight of the sub-evaluation function. In $\text{obdist }(v,\omega )$, it calculates the minimum distance ${d}_{min}$ from each point in the predicted trajectory to the obstacle, which is used to evaluate how far the trajectory is from the obstacle. Trajectories that are farther from the obstacle will get higher scores. $\beta$ is the weight of the sub-evaluation function. In $\text{velocity }(v,\omega )$, the linear velocity and angular velocity are used as the evaluation basis to evaluate the efficiency of reaching the target and the turning situation. A trajectory with a larger linear velocity and a smaller angular velocity will get a higher score, and the relationship between the linear velocity, the angular velocity and the score is linear. $\sigma$ means normalization. Since the dimensions of the three sub-evaluation functions are different, there are distances and radians, and it is meaningless to add them directly. Normalization converts them into the proportion of the total score of similar functions, which can unify the dimension and convert each score value to the same order of magnitude, which is convenient for the subsequent optimization of each weight^27–29^. The normalization of the sub-evaluation function is shown in Eq. (9):

9 $$\begin{array}{c} \, \text{normal\_heading} \, (i)=\frac{\text{ heading }(i)}{\sum_{i=1}^{n} \text{heading}(i)}\\ \, \text{normal\_obdist} \, (i)=\frac{{\text{obdist}}(i)}{\sum_{i=1}^{n} \text{obdist}(i)}\\ \, \text{normal\_velocity} \, (i)=\frac{{\text{velocity}}(i)}{\sum_{i=1}^{n} \text{velocity}(i)}\end{array}$$

**Figure 3:**
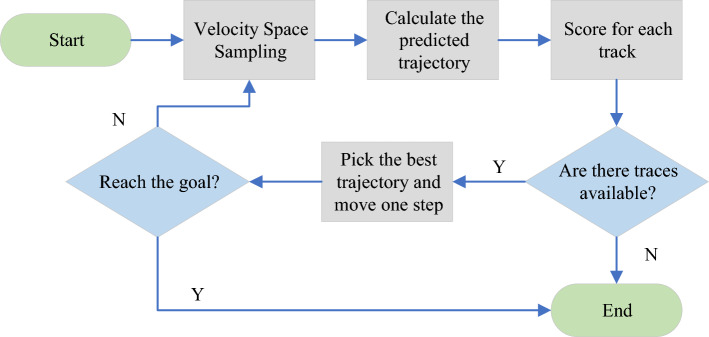
The flow chart of the DWA.

In Eq. (9), $i$ represents the current trajectory, and $n$ is all predicted trajectories. The specific flow chart of the DWA is shown in Fig. 3.

### Improved DWA

There are only three evaluation functions in the traditional DWA, and the adaptability to complex environments is not strong. It is easy to hesitate for a long time in front of obstacles, and to fall into the local optimum. Here, three evaluation functions are analyzed, modified and added to enhance the performance of DWA^30^.

**Figure 4:**
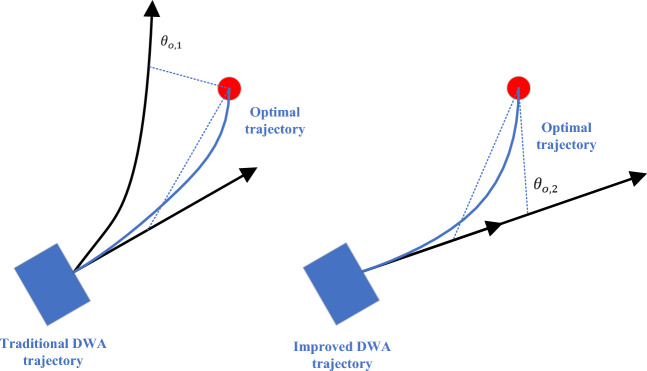
Analysis of $\text{heading }(v,\omega )$ function.

For the $\text{heading }(v,\omega )$ function, to make the predicted path have enough forward-looking, the forward simulation time generally takes dozens of time steps. When the value of $T$ is (10 ~ 30)$\Delta t$, the judgment angle of this function is based on the last position of the predicted trajectory. It means that the position after the robot continues to travel at the current speed after $T$ time, but in fact, the speed of the robot will be reselected after $\Delta t$, that is, the predicted path is only a short distance where the robot will actually go, as shown in Fig. 4.

In Fig. 4, the position and orientation of trajectory 1 after several time steps are more ideal, and the robot should move according to trajectory 1. According to the evaluation method of the original function, the angles will be calculated based on the positions of the end of the two trajectories. It shows from the figure that ${\theta }_{o,2}<{\theta }_{o,1}$, the score of trajectory 2 will be higher than that of trajectory 1, which is inconsistent with the actual situation. If the angle is calculated according to the position after several time steps on the trajectory, it indicates from the figure that ${\theta }_{o,1}<{\theta }_{o,2}$, the score of trajectory 1 is higher than that of trajectory 2, which is consistent with the actual situation. The purpose of this modification is to prevent the position of the calculated angle from being too far from the original position to lose the reference value. The setting of the number of time steps is essentially the setting of the distance from the original position. In practical applications, a distance $d$ should be set^31^. According to the current velocity $v$ of the robot, the time step required to move the distance $d$ is estimated. Generally, when $d$ is relatively small, it can be considered that the robot is moving in a straight line. For estimating the number of time steps is shown in Eq. (10):

10 $${n}_{\Delta t}={\text{fix}}(d,v)$$

When calculating the $\text{heading }(v,\omega )$ score, the angle should be calculated from the position ${n}_{\Delta t}$ time steps later in the predicted trajectory.

**Figure 5:**
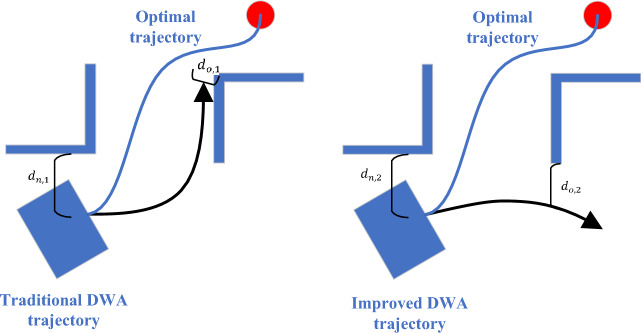
Analysis of $\text{obdist }(v,\omega )$ function.

For the $\text{obdist }(v,\omega )$ function, the two predicted trajectories are compared when the situation shown in Fig. 5 occurs.

Comparing the two predicted trajectories in Fig. 5, it is obvious that the position of trajectory 1 after several time steps is more ideal, and the robot should move according to trajectory 1. According to the evaluation method of the original function, the distance to the obstacle will be calculated from all sampling points from the beginning to the end of the two trajectories. Since ${d}_{o,1}$ is less than the robot's safe distance, the trajectory will be discarded directly. Trajectories with similar trends in trajectory 1 are at risk of being discarded, and trajectories with similar directions as trajectory 2 can obtain higher scores, which is inconsistent with the actual situation. Actually, the $\text{obdist }(v,\omega )$ function has two functions: trajectory scoring and discarding the trajectory. From the trend, trajectory 1 is indeed more likely to collide with obstacles than trajectory 2, but not in a short time, and the orientation of the robot will gradually change, so it is unreasonable to directly discard trajectory 1. The evaluation trajectory of this function does not need to be changed. When the trajectory is discarded, the distance from the obstacle should be calculated from the trajectory from the beginning to the position after several time steps. Figure 5 shows that the lengths of ${d}_{n,1}$ and ${d}_{n,2}$ are similar, and they are both larger than the safe distance to be retained, and the two trajectories can continue to be scored by other functions. This can reduce the negative effect this function may have on some terrain with many obstacles.

**Figure 6:**
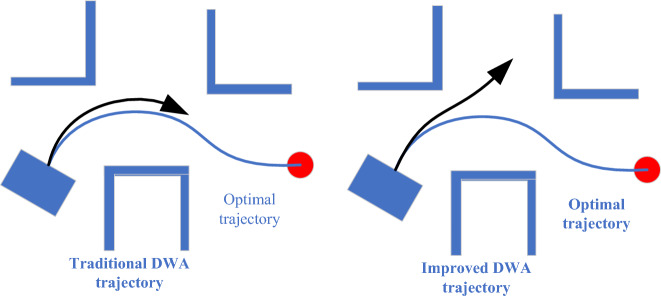
Analysis of $\text{velocity }(v,\omega )$ function.

For the $\text{velocity }(v,\omega )$ function, the score is related to the linear velocity and angular velocity of the trajectory, making the robot move as fast as possible and avoid unnecessary turns. In the original function, the two velocities are independently scored and added, but in some terrains with many obstacle areas, it is more reasonable to zigzag through the smaller linear velocity and the larger angular velocity, as shown in Fig. 6.

**Figure 7:**
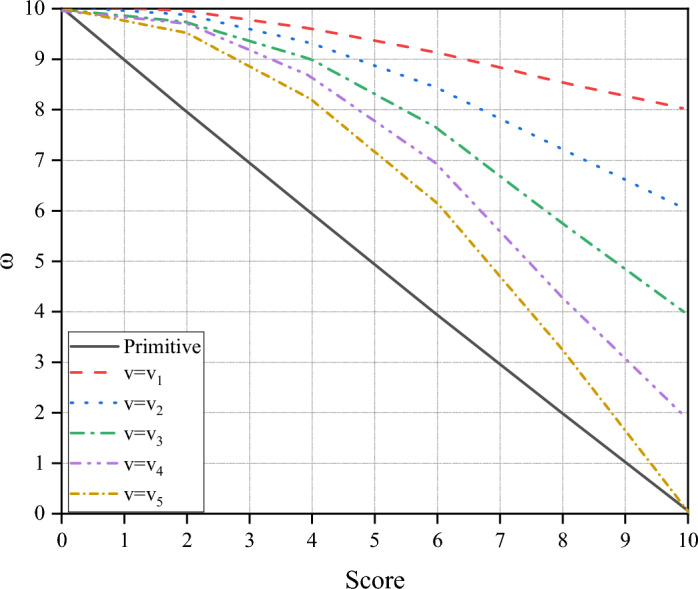
Relationship between angular velocity and score.

The corresponding linear velocities of the two trajectories are the same, and the angular velocity of trajectory 1 is greater than that of trajectory 2. Comparing the two predicted trajectories, it is obvious that trajectory 1 is more ideal. According to the original function, the score of trajectory 2 will be higher than that of trajectory 1, which is inconsistent with the actual situation. Under the same conditions, the higher the linear velocity, the faster the robot can reach the terminus. The scoring standard of the linear velocity does not need to be changed, but the angular velocity is not as small as possible. Considering that the linear velocity of terrain with many obstacles is slow, and the angular velocity will not increase the risk factor when the linear velocity is slow, the modified angle scoring function is shown in Fig. 7.

In Fig. 7, v_1_-v_5_ increase in turn, which can reduce the negative effect that this function may play when it needs to bend at a slow speed.

In addition to analyzing and modifying the above original evaluation functions, it also added two evaluation functions, goaldist $(v,\omega )$ and oscillation $(v,\omega )$ to deal with some special cases.

**Figure 8:**
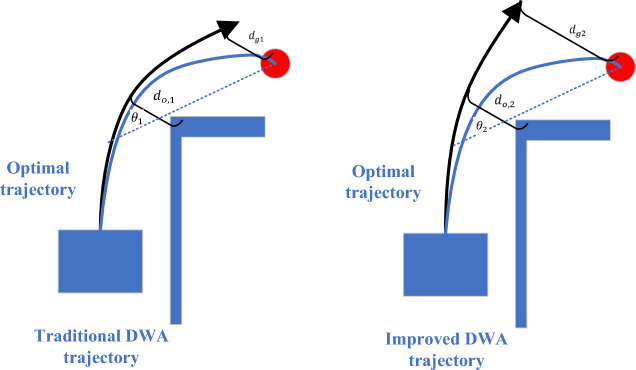
Analysis of ${\text{goaldist}} \;({\text{v,}}\upomega )$ function.

The goaldist $(v,\omega )$ function is added to calculate the minimum distance ${d}_{min}$ from each point in the predicted trajectory to the target point, which is used to evaluate how close the trajectory is to the target point. The trajectory closer to the target point will get a higher score.$\delta$ is the weight of the sub-evaluation function. This function is added mainly to enhance the robot's ability to navigate towards the target point, as shown in Fig. 8.

Comparing the two predicted trajectories, it is obvious that trajectory 1 is more ideal, and the robot should move according to trajectory 1. According to the scoring methods of $\text{heading }(v,\omega )$ and $\text{obdist }(v,\omega )$, ${\theta }_{2}$ is slightly larger than ${\theta }_{1}$. ${d}_{o,2}$ is also greater than ${d}_{o,1}$, and the scores of the two trajectories will be basically the same, making it impossible to accurately judge the pros and cons. After adding the goaldist $(v,\omega )$ function, ${d}_{g1}$ is much smaller than ${d}_{g2}$, so the score of trajectory 1 will be higher than that of trajectory 2, which is in line with the actual situation.

**Figure 9:**
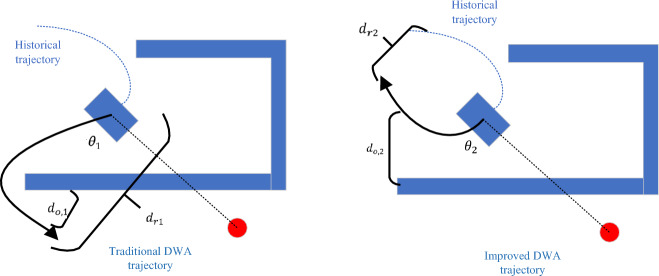
Analysis of ${\text{oscillation}}\;({\text{v,}}\upomega )$ function.

The oscillation $(v,\omega )$ function is added to calculate the distance between the end of the predicted trajectory and the historical trajectory to evaluate the proximity of the trajectory to the historical trajectory. The farther the trajectory is from the historical trajectory, the higher the score will be. $\varepsilon$ is the weight of the sub-evaluation function. This function is added mainly to prevent the robot from going back or spinning in place, as shown in Fig. 9.

Comparing the two predicted trajectories, it is obvious that trajectory 1 is more ideal, and the robot should move according to trajectory 1. According to the scoring methods of $\text{heading }(v,\omega )$ and $\text{obdist }(v,\omega )$, ${\theta }_{2}$ is slightly larger than ${\theta }_{1}$. ${d}_{o,2}$ is also greater than ${d}_{o,1}$, and the scores of the two trajectories will be basically the same, making it impossible to accurately judge the pros and cons. After adding the oscillation $(v,\omega )$ function, ${d}_{r1}$ is much larger than ${d}_{r2}$, so the score of trajectory 1 will be higher than that of trajectory 2, which is in line with the actual situation.

### Simulation experiment

In this simulation experiment, the size of the map is set to 18m*18m, the coordinates of the starting point are (0,0), and the coordinates of the end point are (15,15), and reaching the nearby 0.2m is regarded as reaching the terminus. The safety radius of the robot is 0.5m. The maximum linear velocity is 1m/s. The maximum angular velocity is 2π/3 rad/s. The maximum linear acceleration is 0.2m/s2. the maximum angular acceleration is 2π rad/s2. The velocity resolution in space is 0.01m/s, and the angular velocity resolution is π/90 rad/s.

To enhance the contrast, when simulating the two algorithms, the obstacle map information is set to be the same, and the weight of the evaluation function with the same name is the same. $\text{heading }(v,\omega )$ weight $\alpha$=0.5. ${\text{obdist}} \, (v,\omega )$ weight $\beta$=0.5. $\text{velocity }(v,\omega )$ weight $\gamma$=0.7. In the improved DWA, $\text{heading }(v,\omega )$ moves out the distance $d$=0.5. $\text{obdist }(v,\omega )$ moves out the distance $d$=0.8. goaldist $(v,\omega )$ weight $\delta$=0.5. oscillation $(v,\omega )$ weight $\varepsilon$=0.3. Table 1 shows the parameter settings of the model in this study.

**Table 1:** Parameter settings of the model in this study.

Category	Project	Detailed description/value
Simulation assumptions	Environment type	Closed two-dimensional plane
	Environment size 18 m	18 m * 18 m
	Start and end points	(0,0) to (15,15), arrival at the endpoint is considered within 0.2 m
	Robot safety radius	0.5 m
Numeric examples	Maximum linear velocity	1 m/s
	Maximum angular velocity	"2π/3" rad/s
	Maximum linear acceleration	0.2 m/s^2^
	Maximum angular acceleration	"2π" rad/s^2^
	Velocity resolution	0.01 m/s
	Angular velocity resolution	"π/90" rad/s

As shown in Table 1, in the process of optimizing the DWA, this experiment specifically focuses on three key factors: temporal distance, velocity, and oscillation, making in-depth improvements. Firstly, regarding temporal distance, this study redesigns the sampling strategy of velocity space by introducing finer time interval divisions, thereby enhancing the accuracy of the algorithm in predicting future trajectories. This not only helps robots respond to environmental changes more quickly but also effectively reduces decision-making errors caused by time delays. Secondly, optimizing velocity is also one of the main focuses of this improvement. The study optimizes the velocity sampling range to make it more consistent with actual motion situations, and by scoring trajectories at different speeds, ensures that the robot can select the optimal speed combination during movement. This not only improves the robot's motion efficiency but also enhances its adaptability in different scenarios. Lastly, concerning the oscillation issue, this experiment introduces a new trajectory smoothing algorithm, effectively reducing unnecessary oscillations and shaking by smoothing the robot's motion trajectory. This not only improves the robot's motion stability but also reduces energy consumption and wear, thus prolonging the robot's lifespan. Overall, this study makes substantial improvements to the traditional DWA at the mathematical level. By adding new sampling strategies, optimizing velocity selection, and introducing trajectory smoothing algorithms, the improved DWA demonstrates significant advantages in terms of temporal distance, velocity, and oscillation. These improvements not only enhance the algorithm's performance and stability but also provide readers with clearer explanations and easier-to-understand technical details.

## Results and analysis of simulation experiment

### Results of simulation experiment

**Figure 10:**
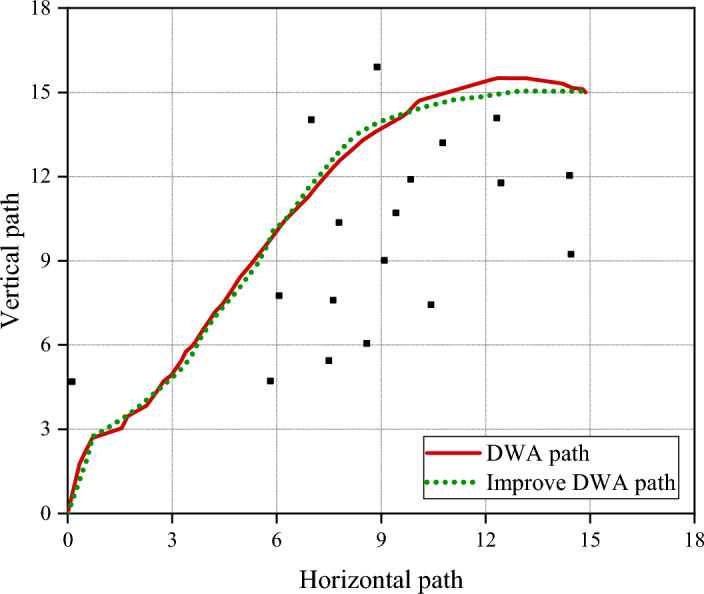
The traditional DWA path and the improved DWA path.

Through simulation experiments, the traditional DWA path and the improved DWA path are finally obtained as shown in Fig. 10.

Figure 10 indicates that the trajectory of the improved algorithm is smooth. Through calculation, the trajectory length of the improved algorithm is 18.7m, and the trajectory length of the original algorithm is 19.4m. The improved algorithm also finds a relatively shorter path.

### Analysis of simulation experiment

**Figure 11:**
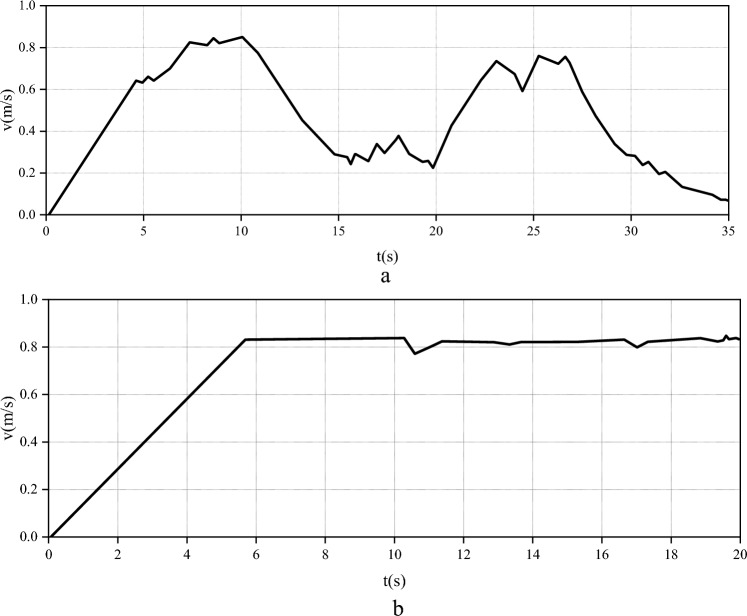
Linear velocity of traditional DWA and improved DWA. (**a**) The linear velocity of the traditional DWA. (**b**) The linear velocity of the improved DWA).

In the experiment, the change of the linear velocity of the robot with time in the traditional DWA and the improved DWA is shown in Fig. 11.

**Figure 12:**
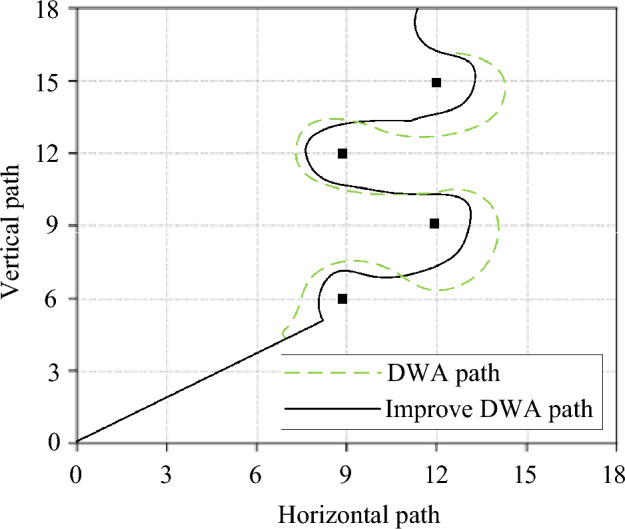
The path design of the robot with the two algorithms under the condition of setting obstacles.

Figure 11 demonstrates that the time to reach the end point of the improved algorithm is 18.6s, while the original algorithm is 34.8s. The improved algorithm significantly shortens the walking time and improves traffic efficiency. The set robot can travel at a constant speed. The speed after encountering an obstacle is compared to reflect the obstacle avoidance status of the robot under different algorithms. Figure 12 reveals the path design of the two algorithm robots under the condition of setting obstacles.

**Figure 13:**
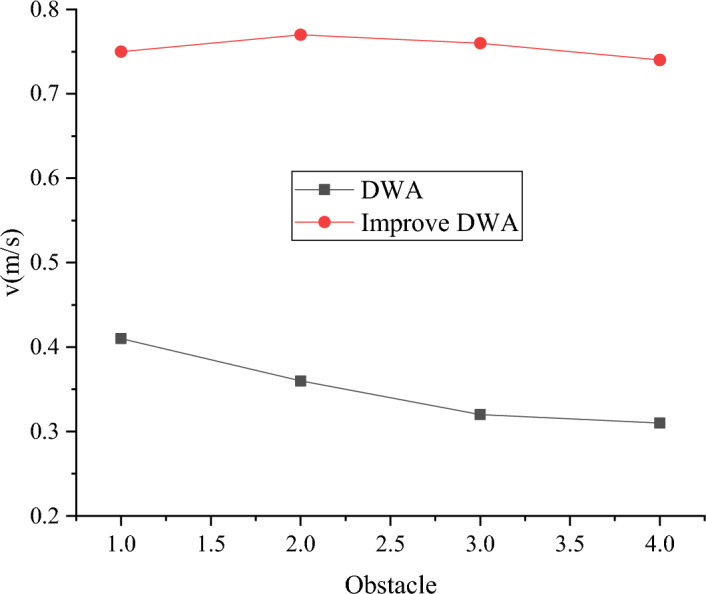
Comparison of the movement speed of the robot to avoid obstacles with different algorithms.

In Fig. 12, the path design of the algorithm robot in the object is basically the same, and the robot chooses a little deviation during the movement process, so the movement speed of the robot of the two algorithms will be different when avoiding obstacles. Figure 13 refers to the comparison of movement speed of the robot of different algorithms.

In Fig. 13, when obstacles are set, the speed of the traditional DWA algorithm robot when passing through obstacles is about 0.4m/s at the highest and 0.3m/s at the lowest. With the increase of obstacles, the movement speed of the robot is getting slower and slower. The average speed of the robot with the improved DWA algorithm is above 0.75m/s, and the overall movement speed of the robot does not change much. The efficiency of the original algorithm is low near obstacles, corners and target points. The main reasons for these are the following three points: (1) In the traditional DWA, the obdist (v,ω) function uses the entire trajectory to judge the distance from the obstacle, which leads to the trajectory of the robot having a larger linear velocity when it approaches the obstacle because of its large length. It is easy to approach or bump into obstacles, score low in this function or be removed directly. (2) In the traditional DWA, the velocity (v, ω) function gives some trajectories with small linear velocity and large angular velocity with low scores, which affects the traffic efficiency of the robot when turning at low speed. (3) In the traditional DWA, the heading (v, ω) function uses the end of the trajectory to determine the heading angle, which leads to the trajectory of the larger linear velocity when the robot approaches the target point, and the angle is large because the end exceeds the target point. Therefore, the score is very low. It can be proved that the proposed improved DWA has strong scientificity and effectiveness. Conducting sensitivity analysis and scientific testing is a crucial step in ensuring the rationality of simulation results. Sensitivity analysis involves adjusting simulation parameters to observe their impact on algorithm performance, aiding in a deeper understanding of algorithm behavior under different conditions. Scientific testing, on the other hand, involves collecting many data samples and employing statistical methods for comparative analysis to validate the effectiveness of algorithm improvements. Such analyses not only enhance the reliability of results but also provide strong support for the stability of algorithms in practical applications. By integrating these analysis results, the performance differences between traditional DWA and improved DWA methods can be more accurately assessed, providing a more reliable and scientific basis for relevant research. Therefore, sensitivity analysis and scientific testing are essential during the analysis of simulation results. Table 2 shows the results of sensitivity analysis and scientific testing.

**Table 2:** Results of sensitivity analysis and scientific testing.

Model type	Average speed (m/s)	Speed variation range (m/s)	Maximum speed when passing obstacles (m/s)	Minimum speed when passing obstacles (m/s)	Speed variation trend with increased obstacles	Efficiency evaluation	Significance test t-value	Significance test df	Significance test p-value
Traditional DWA	0.55	0.3–0.4	0.4	0.3	Slowing down	Inefficient	8.56	198	< 0.01
Improved DWA	0.75	0.70–0.80	0.78	0.72	Stable	Efficient			

In Table 2, the improved DWA model exhibits significant advantages over the traditional DWA model in the simulation experiments. Firstly, the average speed of the improved DWA model is higher, and the range of speed variation is smaller, indicating that its motion is more stable and efficient. Secondly, when facing obstacles, the improved DWA model can maintain a higher speed through them, while the traditional model experiences a significant decrease in speed, demonstrating the superiority of the improved model in dealing with complex environments. Additionally, the results of the significance test further confirm that this performance difference is significant. Overall, the improved DWA model surpasses the traditional model in terms of speed, stability, and obstacle handling ability, providing a more reliable and efficient solution for robot navigation, with broad prospects for application.

## Conclusion

The regulation of pedestrian flow is studied by improving DWA. Firstly, the pedestrian traffic flow and the traditional DWA are deeply researched. Secondly, the traditional DWA is improved, which has weak adaptability to the environment and is easy to fall into the local optimum. The goaldist $(v,\omega )$ function and the oscillation $(v,\omega )$ function is added on the basis of the original three evaluation functions. An improved DWA function is proposed. Finally, the traditional DWA and the improved DWA are compared through simulation experiments. The experimental map is 18m*18m. The trajectory length of the robot of the traditional algorithm is 19.4m, and the trajectory length of the robot of the improved DWA is 18.7m. Compared with the traditional algorithm, the trajectory distance obtained by the improved DWA is shorter. Moreover, the improved time of 18.6s is far less than the 34.8s of the original algorithm, and the traffic efficiency is higher. Experiments show that the proposed improved DWA is scientific and effective. In the path planning experiment, due to limited capabilities, the limitation of DWA sampling space and the limitation of lidar accuracy, the robot has limited ability to detect and avoid dynamic obstacles, and its recovery behavior also needs to be realized^32^. It needs to be supplemented with visual information and develop a more effective obstacle avoidance algorithm, which will be improved in the future. It has certain reference significance for the improvement of DWA and the regulation of pedestrian flow.

## Data Availability

The datasets used and/or analysed during the current study available from the corresponding author on reasonable request.
